# Crystal structure of an indium–salicyl­hydroximate complex cation: [In_4_(H_2_shi)_8_(H_2_O)_6_](NO_3_)_4_·8.57H_2_O

**DOI:** 10.1107/S2056989022007964

**Published:** 2022-08-18

**Authors:** Ozha A. Aziz, Matthias Zeller, Curtis M. Zaleski

**Affiliations:** a Department of Chemistry and Biochemistry, Shippensburg University, Shippensburg, PA 17257, USA; b Department of Chemistry, Purdue University, West Lafayette, IN 47907, USA; Tulane University, USA

**Keywords:** indium, salicyl­hydroxamic acid, crystal structure

## Abstract

The synthesis and crystal structure for the title compound, hexa­aqua­hexa­kis(μ-2-hy­droxy­benzene­carbo­hydrox­a­mato)bis­(2-hy­droxy­benzene­carbo­hydrox­a­m­ato)tetra­indium(III) tetra­nitrate 8.57-hydrate + unknown solvent, [In_4_(H_2_shi)_8_(H_2_O)_6_](NO_3_)_4_·8.57H_2_O·solvent, where H_2_shi^−^ is salicylhydrox­imate (C_7_H_5_NO_3_), are reported. The complex cation of the structure, [In_4_(H_2_shi)_8_(H_2_O)_6_]^4+^, is a dimer with a step-like topology and possesses an inversion center that relates each [In_2_(H_2_shi)_4_(H_2_O)_3_]^2+^ side of the complex cation. Each In^III^ ion is seven-coordinate with a penta­gonal–bipyramidal geometry, and the salicyl­hydroximate ligands have a 1− charge as only the oxime oxygen of the ligand is deprotonated. Four inter­stitial nitrate anions maintain the charge balance of the compound. One of the nitrate anions (and its symmetry equivalent) is disordered over two different orientations with an occupancy ratio of 0.557 (7) to 0.443 (7). The inter­stitial solvent water mol­ecules show substantial disorder. Approximately 8.57 water mol­ecules per formula unit were refined as disordered and partially occupied, while a suitable model could not be devised for the other extensively disordered solvent mol­ecules (water and possibly methanol as this was the synthesis solvent). Thus, these latter solvent mol­ecules were instead treated with the SQUEEZE routine [Spek (2015). *Acta Cryst*. C**71**, 9–18.] as implemented in the program *PLATON*, and the procedure corrected for 151 electrons within solvent-accessible voids of 367 Å^3^.

## Abstract 2

The title compound [In_4_(H_2_shi)_8_(H_2_O)_6_](NO_3_)_4_·8.57H_2_O·solvent, where H_2_shi^−^ is salicyl­hydroximate, is a dimer of two [In_2_(H_2_shi)_4_(H_2_O)_3_]^2+^ units that are related by an inversion center. The overall configuration of the dimer has a step-like topology, and each In^III^ ion is seven coordinate with a penta­gonal–bipyramidal geometry.

## 1. Chemical context

Salicyl­hydroxamic acid (H_3_shi) has proven to be a versatile ligand for the class of inorganic macrocyclic coordination compounds known as metallacrowns (MC) (Mezei *et al.*, 2007 ▸). Metallacrowns are the inorganic analogue of organic crown ethers (Pedersen, 1967 ▸). As crown ethers have a carbon–carbon–oxygen ring repeat unit, metallacrowns have a metal–nitro­gen–oxygen repeat unit about the ring of the metallamacrocycle. In addition, as crown ethers, metallacrowns are capable of capturing a metal ion in the central cavity of the structure. Salicyl­hydroxamic acid in its triply deprotonated state (shi^3−^) was used in the synthesis of the first metallacrown, a vanadium-based 9-MC-3 (Pecoraro, 1989 ▸), and since then it has been used to construct numerous MCs including other 9-MC-3 (Lah *et al.*, 1989 ▸), 12-MC-4 (Lah & Pecoraro, 1989 ▸), and 15-MC-5 (Kessisoglou *et al.*, 1994 ▸) compounds. Initially salicyl­hydroxamic acid was mainly used in conjunction with transition-metal ions in both the ring and central metal positions of the metallacrown structure. However, in 2014 our group demonstrated that 12-MC-4 compounds with a salicyl­hydroximate (shi^3−^) framework could incorporate lanthanide ions in the central cavity while using Mn^III^ ions in the ring positions of the MC (Azar *et al.*, 2014 ▸). These mol­ecules proved to be mol­ecular magnets with magnetic behavior consistent with single-mol­ecule magnetism (Boron *et al.*, 2016 ▸). Since then the manganese(III) ions have been replaced with the main-group metals gallium(III) and aluminum(III), and both the lanthanide-gallium (Chow *et al.*, 2016 ▸) and lanthanide-aluminum (Eliseeva *et al.*, 2022 ▸) 12-MC-4 structures with the shi^3−^ ligand are highly luminescent materials in the visible and near-infrared regions. To further explore the chemistry of H_3_shi with other main-group metals, we decided to react the ligand with indium(III), a fellow Group 13 metal. 
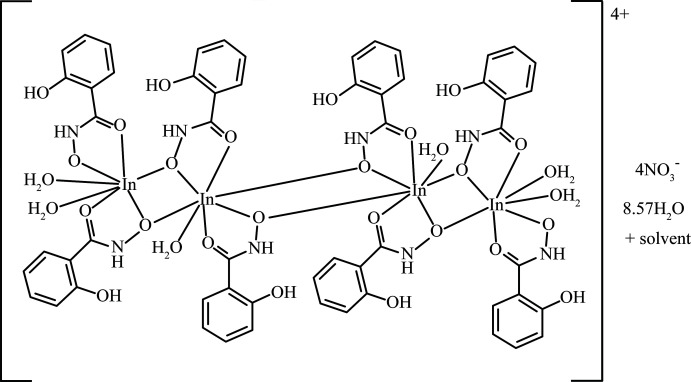


Indium is an appealing target for metallacrown and metallacrown-like compounds, as indium coordination complexes have applications in both the medicinal and material chemistry fields. The radioisotope indium-111 emits gamma radiation and has a half-life of ∼2.8 days. Numerous coordination complexes of the radiometal have been evaluated as potential imaging agents and radiolabels (Pecoraro *et al.*, 1982 ▸; Liu *et al.*, 2003 ▸; Nishikawa *et al.*, 2003 ▸; Ramogida *et al.*, 2015 ▸; Choudhary *et al.*, 2019 ▸). In addition, indium coordination complexes have been investigated as precursors for indium oxide thin films (Xu *et al.*, 2000 ▸; Chou *et al.*, 2003 ▸; Lee *et al.*, 2018 ▸; Yoo *et al.*, 2021 ▸) and as luminophores (Lee *et al.*, 2017 ▸). Herein we report the synthesis and single-crystal X-ray crystal structure of [In_4_(H_2_shi)_8_(H_2_O)_6_](NO_3_)_4_·8.57H_2_O·solvent, **1**, where H_2_shi^−^ is the singly deprotonated version of salicyl­hydroxamic acid. Future work will focus on the potential use of the compound for radiopharmacological or thin film applications.

## 2. Structural commentary

**Figure 1:**
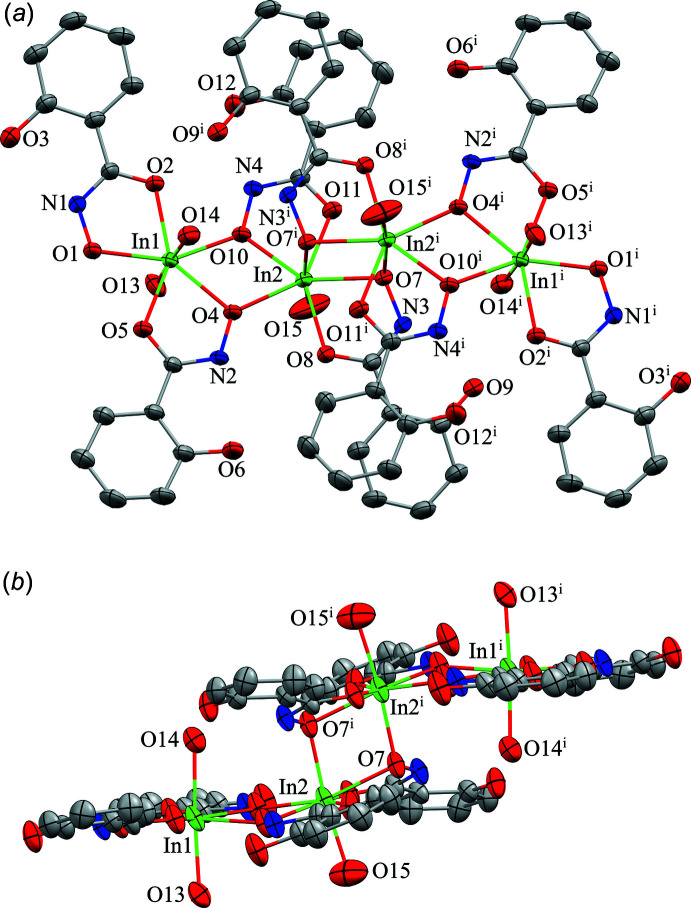
The single-crystal X-ray structure of [In_4_(H_2_shi)_8_(H_2_O)_6_](NO_3_)_4_·8.57H_2_O·solvent, **1**, with displacement ellipsoids at the 50% probability level [symmetry code: (i) −*x* + 1, −*y* + 1, −*z* + 1]. (*a*) top view with only the metal ions and heteroatoms labeled for clarity and (*b*) side view with only the metal ions and axial heteroatoms labeled. In addition, hydrogen atoms, inter­stitial nitrate anions, inter­stitial water mol­ecules, and disorder have been omitted for clarity. Color scheme: green – In, red – oxygen, dark blue – nitro­gen, and gray – carbon. All figures were generated with the program *Mercury* (Macrae *et al.*, 2020 ▸).

**Figure 2:**
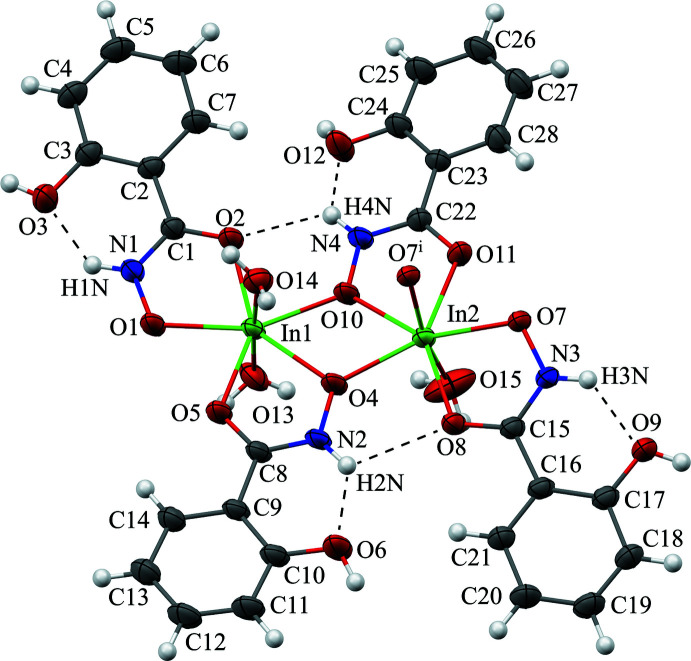
Top view of a [In_2_(H_2_shi)_4_(H_2_O)_3_]^2+^ unit of **1** with displacement ellipsoids at the 50% probability level [symmetry code: (i) −*x* + 1, −*y* + 1, −*z* + 1]. In addition, the intra­molecular hydrogen bonding in **1** between the hydrogen atoms (white) of the oxime nitro­gen atoms and the phenol oxygen atoms and between the hydrogen atoms of the oxime nitro­gen atoms and the carbonyl oxygen atoms are displayed. See Fig. 1 ▸ for additional display details.

Compound **1**, [In_4_(H_2_shi)_8_(H_2_O)_6_](NO_3_)_4_·8.57H_2_O·solvent, consists of four indium ions with a 3+ charge (total 12+ charge) that is counterbalanced by eight singly deprotonated salicyl­hydroximate anions (H_2_shi^−^) and four inter­stitial nitrate ions (total 12− charge). Only the oxime oxygen atoms (O1, O4, O7, and O10) of the H_2_shi^−^ ligands are deprotonated. The complex cation structure of **1**, [In_4_(H_2_shi)_8_(H_2_O)_6_]^4+^, is a dimer with a step-like topology (Fig. 1 ▸). The dimer features four In^III^ ions in a chain {In1, In2, In2^i^, In1^i^; [symmetry code: (i) −*x* + 1, −*y* + 1, −*z* + 1]} and each half of the dimer is related by an inversion center located between the two central indium ions (In2) (Fig. 1 ▸). Each side of the dimer contains two indium(III) ions, four H_2_shi^−^ anions, and three water mol­ecules: [In_2_(H_2_shi)_4_(H_2_O)_3_]^2+^ (Fig. 2 ▸). Each half of the complex cation is connected *via* the middle In2 centers where two oxime oxygens of symmetry-related H_2_shi^−^ ligands bind to both In2 ions. Both In^III^ ions are seven-coordinate with penta­gonal–bipyramidal geometry (Table 1 ▸; Figs. 1 ▸ and 2 ▸). The geometry was determined with the program *SHAPE 2.1* (Llunell *et al.*, 2013 ▸; Pinsky & Avnir, 1998 ▸; Casanova *et al.*, 2004 ▸). For In1, the penta­gonal plane consists of five oxygen atoms from three different H_2_shi^−^ ligands. Two of the ligands bind in a bidentate fashion using both the oxime and carbonyl oxygen atoms of the ligand to form two five-membered chelate rings about the In^III^ center. The third H_2_shi^−^ binds in a monodentate fashion *via* the oxime oxygen atom. The axial positions of the coordination geometry are occupied by two water mol­ecules. In1 is connected to In2 *via* two bridging oxime oxygen atoms. For In2, the metal center binds to four H_2_shi^−^ anions, three from one half of the dimer and one from the symmetry-related portion of the cation. Two of the H_2_shi^−^ ligands bind in a bidentate fashion with oxime and carbonyl oxygen atoms and form two five-membered chelate rings, while the other two bind in a monodentate fashion *via* the oxime oxygen atoms. Three of the H_2_shi^−^ anions (two bidentate and one monodentate) provide the five oxygen atoms of the penta­gonal plane. The axial direction consists of one water mol­ecule and one oxime oxygen atom. This axial oxime oxygen atom then binds to the symmetry equivalent In2 ion of the other portion of the cation and thus generates the step-like topology where each half of the dimer consists of two In^III^ ions (Fig. 1 ▸ *b*).

The inter­stitial area contains an ordered nitrate anion and a nitrate anion that is disordered over two positions with an occupancy ratio of 0.557 (7) to 0.443 (7). In addition, several solvent water mol­ecules (*ca* 8.57 per formula unit) were found to be disordered, and when possible the disorder and hydrogen bonding were refined. All of the inter­stitial water mol­ecules are only partially occupied (associated with O22–O27) and due to the large amount of disorder, no attempts were made to match occupancies. In addition, some solvent mol­ecules (water and/or methanol) were found to have excessive disorder and a suitable model could not be devised. These solvent mol­ecules were instead augmented with the SQUEEZE routine (Spek, 2015 ▸) as implemented in the program PLATON (Spek, 2022 ▸). Complete details regarding the SQUEEZE results can be found in the *Refinement* section.

## 3. Supra­molecular features

For the [In_4_(H_2_shi)_8_(H_2_O)_6_]^4+^ cation of **1**, several intra­molecular hydrogen bonds exist between the protonated oxime nitro­gen atoms (N1, N2, N3, and N4) of the H_2_shi^−^ ligands and the protonated phenol oxygen atoms (O3, O6, O9, and O12, respectively) of the same ligand with the hydrogen atom of the nitro­gen atom bonding to the oxygen atom (Fig. 2 ▸; Table 2 ▸). In addition, the hydrogen atom of the oxime nitro­gen atom (N2 and N4) also forms a hydrogen bond to the carbonyl oxygen atom (O8 and O2, respectively) of a neighboring H_2_shi^−^ ligand (Fig. 2 ▸), and the hydrogen atom of the oxime nitro­gen atom (N3) bonds to the oxygen atom (O14) of the water mol­ecule coordinated to In1.

**Figure 3:**
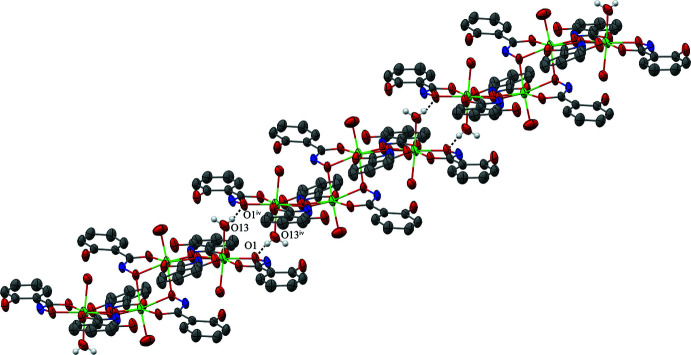
Inter­molecular hydrogen bonding in **1** between the hydrogen atom (white) of the water mol­ecule associated with O13 (coordinated to In1) and the oxime oxygen atom (O1) of a neighboring complex cation of **1** [symmetry code: (iv) −*x* + 1, −*y* + 1, −*z*]. The hydrogen bonding results in a one-dimensional chain. For clarity only the oxygen atoms involved in the hydrogen bonding have been labeled. See Fig. 1 ▸ for additional display details.

There is one inter­molecular hydrogen bond between neighboring complex cations of **1** (Table 2 ▸). The hydrogen atom of the water mol­ecule (associated with O13) coordinated to In1 forms a hydrogen bond to an oxime oxygen atom (O1) of a neighboring complex cation. In addition, the reciprocal hydrogen bond is also formed between the two cations. Due to the inversion center of the complex cation, these hydrogen bonds occur on both sides of the [In_4_(H_2_shi)_8_(H_2_O)_6_]^4+^ ion; thus, a one-dimensional chain of the dimers is generated (Fig. 3 ▸).

Furthermore, several inter­molecular hydrogen bonds exist between the partially occupied inter­stitial water mol­ecules (O22–O27) themselves and between the inter­stitial water mol­ecules and the protonated phenol oxygen atoms (O3 and O6) and the carbonyl oxygen atoms (O5 and O11) atoms of the H_2_shi^−^ ligands, the water mol­ecules (O13–O15) coord­inated to the In^III^ ions, and the oxygen atoms (O18 and O21) of the inter­stitial nitrate anions (Table 2 ▸). Lastly, the protonated phenol oxygen atoms (O6, O9, and O12) of the H_2_shi^−^ ligands form hydrogen bonds to the oxygen atoms (O16, O17, O18, and O20) of inter­stitial nitrate ions, and the coordinated water mol­ecules (O14 and O15) form hydrogen bonds with the oxygen atoms (O19, O20, and O21) of inter­stitial nitrate ions.

## 4. Database survey

A survey of the Cambridge Structural Database (CSD version 5.43, update March 2022; Groom *et al.*, 2016 ▸) lists only two other structures with indium bound to hydroxamic acid ligands, though neither are salicyl­hydroxamic acid. One structure (JAGWUJ; Matsuba *et al.*, 1988 ▸) contains an indium(III) ion bound to three benzo­hydroximate ligands in an octa­hedral propeller coordination geometry with Δ configuration. The other structure (VOLNIU; Seitz *et al.*, 2008 ▸) is an indium(III) ion in a trigonal prismatic coordination geometry bound to a tripodal ligand based on 1-oxo-2-hy­droxy-iso­quinoline-3-carb­oxy­lic acid, an aromatic hydroxamic acid. In addition, there are five other di-metallic structures [DEYSIM (Lee *et al.*, 2018 ▸); UWOFIY, UWOFOE, UWOGAR, UWOGEV (Yoo *et al.*, 2021 ▸)] of indium bound to *N*-alk­oxy carboxamide ligands. This class of ligands is closely related to hydroxamic acids as they also have a O–C–N–O connectivity, but the oxygen atom attached to the nitro­gen atom is bound to an alkyl group instead of being an acidic hydrogen atom.

## 5. Synthesis and crystallization

**Synthetic Materials**

Salicyl­hydroxamic acid (H_3_shi, >98%) was purchased from TCI America. Indium(III) nitrate hydrate (99.999%-In; Puratrem) was purchased from Strem Chemicals. Methanol (ACS grade) was purchased from VWR Chemicals BDH. All reagents were used as received and without further purification.

**Synthesis [In_4_(H_2_shi)_8_(H_2_O)_6_](NO_3_)_4_·8.57H_2_O·solvent**, **1.** Salicyl­hydroxamic acid (1 mmol) was dissolved in 10 mL of methanol resulting in a clear, light-pink solution. In a separate beaker, indium(III) nitrate hydrate (1 mmol; with an assumption of five waters of hydration) was dissolved in 10 mL of methanol resulting in a clear, colorless solution. The two solutions were mixed resulting in a clear, slightly pink solution and then allowed to stir overnight. The solution was then filtered, and no solid was recovered. The filtrate remained clear and slightly pink. X-ray quality clear and colorless crystals were grown in 21 days by slow evaporation of the solvent. The percentage yield was 43% based on salicyl­hydroxamic acid. FT–IR bands (ATR, cm^−1^): 1604, 1566, 1519, 1486, 1451, 1336, 1311, 1241, 1152, 1099, 1064, 1035, 927, 858, 821, 813, 770, 746, 665, 588, 560.

## 6. Refinement

A nitrate ion (associated with N5) was refined as disordered. The two disordered moieties were restrained to have similar geometries as the ordered nitrate ion (SAME command of *SHELXL*, first and second esds were 0.02 and 0.04 Å). *U*_ij_ components of ADPs for disordered atoms closer to each other than 2.0 Å were restrained to be similar (SIMU command of *SHELXL*, first and second esds were 0.01 and 0.02 Å^2^). Subject to these conditions the occupancy ratio refined to 0.443 (7) to 0.557 (7).

Solvate mol­ecules were found to be disordered. For the better defined solvate mol­ecules, distances to potential hydrogen-bond acceptors indicated these mol­ecules to be water (methanol was used as the crystallization solvent and waters of hydration were present in the starting materials used) and these were refined as disordered water mol­ecules. For mol­ecules not directly hydrogen bonded to the main mol­ecule, disorder was found to be excessive (greater than three- to fourfold disorder of water and/or methanol) and no suitable model could be devised. The structure factors associated with the disordered solvate mol­ecules were instead augmented *via* reverse Fourier transform methods using the SQUEEZE routine (Sluis & Spek, 1990 ▸; Spek, 2015 ▸) as implemented in the program *PLATON* (Spek, 2020 ▸). The resultant FAB file containing the structure-factor contribution from the electron content of the void space was used together with the original hkl file in the further refinement. (The FAB file with details of the SQUEEZE results is appended to the CIF file). The SQUEEZE procedure corrected for 151 electrons within solvent-accessible voids of 367 Å^3^.

Resolved disordered water mol­ecules were assigned occupancy values. For ‘outlying’ water mol­ecules occupancies did not refine to full occupancy for each site (due to excessive disorder, or part of the site overlapping with squeezed areas) and no attempts were made to match the occupancies of these water mol­ecules with other moieties in the structure to add up to unity for each site. *U*_ij_ components of ADPs for disordered atoms closer to each other than 2.0 Å were restrained to be similar [SIMU command of *SHELXL*, first and second esds were 0.01 and 0.02 (O22, O22*B* O24*B*, O25) or 0.001 (O26, O27, O26*B*, O27*B*) Å^2^]. Water hydrogen-atom positions were initially refined and O—H and H⋯H distances were restrained to 0.84 (2) and 1.36 (2) Å, respectively, while a damping factor was applied. Some water hydrogen-atom positions were further restrained based on hydrogen-bonding considerations. In the final refinement cycles, hydrogen atoms with low occupancies were constrained to ride on their carrier atoms and the damping factor was removed. Subject to these conditions, the occupancy rates refined to the values given in the tables of the CIF. Additional crystal data, data collection, and structure refinement details are summarized in Table 3 ▸.

## Supplementary Material

Crystal structure: contains datablock(s) I, global. DOI: 10.1107/S2056989022007964/mw2189sup1.cif

Structure factors: contains datablock(s) I. DOI: 10.1107/S2056989022007964/mw2189Isup2.hkl

CCDC reference: 2195615

Additional supporting information:  crystallographic information; 3D view; checkCIF report

## Figures and Tables

**Table 1:** Continuous Shapes Measures (CShM) values for the geometry about the seven-coordinate In^III^ ions of **1**.

Shape	In1	In2
Heptagon (*D*_7*h*_)	33.951	33.043
Hexagonal pyramid (*C*_6*v*_)	25.413	23.853
Penta­gonal bipyramid (*D*_5*h*_)	0.290	1.046
Capped octa­hedron (*C*_3*v*_)	7.067	5.351
Capped trigonal prism (*C*_2*v*_)	5.268	4.128
Johnson penta­gonal bipyramid (*J*13; *D*_5*h*_)	3.798	4.596
Johnson elongated triangular pyramid (*J*7, *C*_3*v*_)	22.464	21.243

**Table 2:** Hydrogen-bond geometry (Å, °)

*D*—H⋯*A*	*D*—H	H⋯*A*	*D*⋯*A*	*D*—H⋯*A*
O3—H3*O*⋯O22^i^	0.84	1.83	2.664 (4)	171
O3—H3*O*⋯O22*B* ^i^	0.84	1.97	2.668 (17)	140
O6—H6*O*⋯O18^ii^	0.84	1.98	2.775 (10)	157
O6—H6*O*⋯O17*B* ^ii^	0.84	2.06	2.810 (5)	149
O9—H9*O*⋯O20^iii^	0.84	1.88	2.717 (3)	176
O12—H12*O*⋯O17	0.84	1.99	2.778 (5)	156
O12—H12*O*⋯O16*B*	0.84	1.81	2.609 (6)	158
O13—H13*B*⋯O24	0.82 (2)	2.09 (3)	2.761 (6)	140 (4)
O13—H13*B*⋯O25	0.82 (2)	2.01 (4)	2.64 (3)	134 (4)
O13—H13*B*⋯O24*B*	0.82 (2)	1.70 (2)	2.485 (7)	160 (5)
O13—H13*A*⋯O1^iv^	0.84 (2)	1.77 (2)	2.601 (2)	170 (4)
O14—H14*A*⋯O26	0.86 (2)	1.83 (2)	2.631 (5)	154 (4)
O14—H14*A*⋯O26*B*	0.86 (2)	1.82 (2)	2.629 (8)	158 (4)
O14—H14*B*⋯O19^i^	0.84 (2)	1.99 (2)	2.782 (3)	157 (4)
O15—H15*A*⋯O25	0.84 (2)	2.11 (3)	2.93 (3)	171 (6)
O15—H15*A*⋯O24*B*	0.84 (2)	2.10 (3)	2.891 (9)	158 (5)
O15—H15*B*⋯O20	0.83 (2)	2.27 (3)	2.970 (4)	142 (5)
O15—H15*B*⋯O21	0.83 (2)	2.16 (3)	2.924 (4)	154 (6)
O23—H23*B*⋯O5	0.89	2.16	3.003 (6)	160
O22—H22*A*⋯O18^v^	0.82 (2)	2.11 (4)	2.842 (12)	148 (6)
O22—H22*B*⋯O21^vi^	0.82 (2)	2.03 (3)	2.832 (5)	164 (7)
O24—H24*A*⋯O25	0.86 (2)	2.03 (2)	2.80 (3)	149 (6)
O24—H24*B*⋯O21	0.85 (2)	2.21 (4)	3.003 (5)	155 (6)
O22*B*—H22*C*⋯O21^vi^	0.84	2.00	2.808 (16)	159
O24*B*—H24*C*⋯O22*B*	0.85 (2)	2.02 (2)	2.824 (19)	156 (7)
O26—H26*A*⋯O27	0.83 (2)	1.56 (6)	2.259 (16)	140 (9)
O27—H27*B*⋯O23	0.86	2.14	2.987 (19)	168
O26*B*—H26*C*⋯O27*B*	0.84	1.73	2.31 (2)	124
O26*B*—H26*D*⋯O23	0.84	2.38	3.033 (13)	134
O27*B*—H27*D*⋯O11^vii^	0.85	2.13	2.955 (18)	163
N1—H1*N*⋯O3	0.88	1.92	2.605 (3)	134
N2—H2*N*⋯O6	0.88	2.02	2.669 (2)	130
N2—H2*N*⋯O8	0.88	2.48	2.935 (3)	113
N3—H3*N*⋯O9	0.88	1.94	2.605 (3)	132
N3—H3*N*⋯O14^vii^	0.88	2.24	2.939 (3)	137
N4—H4*N*⋯O2	0.88	2.33	2.804 (3)	114
N4—H4*N*⋯O12	0.88	1.97	2.621 (3)	130

Symmetry codes: (i) 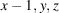 ; (ii) 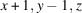 ; (iii) 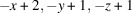 ; (iv) 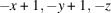 ; (v) 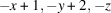 ; (vi) 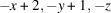 ; (vii) 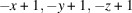 .

**Table 3:** Experimental details

Crystal data	
Chemical formula	[In_4_(C_7_H_6_NO_3_)_8_(H_2_O)_6_](NO_3_)_4_·8.57H_2_O·[+solvent]
*M*_r_	2186.80
Crystal system, space group	Triclinic, *P* 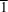
Temperature (K)	150
*a*, *b*, *c* (Å)	11.8435 (5), 14.2195 (6), 14.6918 (5)
α, β, γ (°)	81.905 (2), 70.768 (2), 76.516 (2)
*V* (Å^3^)	2266.22 (16)
*Z*	1
Radiation type	Mo *K*α
μ (mm^−1^)	1.11
Crystal size (mm)	0.17 × 0.16 × 0.06

Data collection	
Diffractometer	Bruker D8 Quest
Absorption correction	Multi-scan (*SADABS*; Krause *et al.*, 2015 ▸)
*T*_min_, *T*_max_	0.680, 0.747
No. of measured, independent and observed [*I* > 2σ(*I*)] reflections	118064, 17348, 12266
*R*_int_	0.049
(sin θ/λ)_max_ (Å^−1^)	0.771

Refinement	
*R*[*F* ^2^ > 2σ(*F* ^2^)], *wR*(*F* ^2^), *S*	0.037, 0.109, 1.02
No. of reflections	17348
No. of parameters	694
No. of restraints	215
H-atom treatment	H atoms treated by a mixture of independent and constrained refinement
Δρ_max_, Δρ_min_ (e Å^−3^)	1.11, −1.49

Computer programs: *APEX4* and *SAINT* (Bruker, 2022 ▸), *SHELXT* (Sheldrick, 2015*a* ▸), *SHELXL2018/3* (Sheldrick, 2015*b* ▸), *ShelXle* (Hübschle *et al.*, 2011 ▸), *Mercury* (Macrae *et al.*, 2020 ▸), and *publCIF* (Westrip, 2010 ▸).

## supplementary crystallographic information

### Crystal data

**Table:**

[In_4_(C_7_H_6_NO_3_)_8_(H_2_O)_6_](NO_3_)_4_·8.57H_2_O·[+solvent]	*Z* = 1
*M**_r_* = 2186.80	*F*(000) = 1099
Triclinic, *P*1	*D*_x_ = 1.603 Mg m^−^^3^
*a* = 11.8435 (5) Å	Mo *K*α radiation, λ = 0.71073 Å
*b* = 14.2195 (6) Å	Cell parameters from 9643 reflections
*c* = 14.6918 (5) Å	θ = 2.7–33.1°
α = 81.905 (2)°	µ = 1.11 mm^−^^1^
β = 70.768 (2)°	*T* = 150 K
γ = 76.516 (2)°	Plate, colourless
*V* = 2266.22 (16) Å^3^	0.17 × 0.16 × 0.06 mm

### Data collection

**Table:**

Bruker D8 Quest diffractometer	17348 independent reflections
Radiation source: fine focus sealed tube X-ray source	12266 reflections with *I* > 2σ(*I*)
Triumph curved graphite crystal monochromator	*R*_int_ = 0.049
Detector resolution: 7.4074 pixels mm^-1^	θ_max_ = 33.2°, θ_min_ = 2.7°
ω and phi scans	*h* = −18→18
Absorption correction: multi-scan (SADABS; Krause *et al.*, 2015)	*k* = −21→21
*T*_min_ = 0.680, *T*_max_ = 0.747	*l* = −22→22
118064 measured reflections	

### Refinement

**Table:**

Refinement on *F*^2^	215 restraints
Least-squares matrix: full	Hydrogen site location: mixed
*R*[*F*^2^ > 2σ(*F*^2^)] = 0.037	H atoms treated by a mixture of independent and constrained refinement
*wR*(*F*^2^) = 0.109	*w* = 1/[σ^2^(*F*_o_^2^) + (0.0494*P*)^2^ + 2.2183*P*] where *P* = (*F*_o_^2^ + 2*F*_c_^2^)/3
*S* = 1.02	(Δ/σ)_max_ = 0.001
17348 reflections	Δρ_max_ = 1.11 e Å^−^^3^
694 parameters	Δρ_min_ = −1.48 e Å^−^^3^

### Special details

**Table:**

Geometry. All esds (except the esd in the dihedral angle between two l.s. planes) are estimated using the full covariance matrix. The cell esds are taken into account individually in the estimation of esds in distances, angles and torsion angles; correlations between esds in cell parameters are only used when they are defined by crystal symmetry. An approximate (isotropic) treatment of cell esds is used for estimating esds involving l.s. planes.
Refinement. A nitrate ion was refined as disordered. The two disordered moieties were restrained to have similar geometries. Uij components of ADPs for disordered atoms closer to each other than 2.0 Angstrom were restrained to be similar. Subject to these conditions the occupancy ratio refined to 0.443 (7) to 0.557 (7). Solvate water molecules were found to be disordered. Where possible, disorder was refined. For molecules not directly hydrogen bound to the main molecule disorder was found to be excessive (> 3-4 fold disorder of water and/or methanol) and no suitable model that could be devised. The structure factors associated with the disordered solvate molecules were instead augmented via reverse Fourier transform methods using the SQUEEZE routine (P. van der Sluis & A.L. Spek (1990). Acta Cryst. A46, 194-201) as implemented in the program Platon. The resultant FAB file containing the structure factor contribution from the electron content of the void space was used in together with the original hkl file in the further refinement. (The FAB file with details of the Squeeze results is appended to this cif file). The Squeeze procedure corrected for 151 electrons within solvent accessible voids of 367 Angstrom cubed.Resolved disordered water molecules were assigned occupancy rates. For "outlying" water molecules occupancies did not refine to full occupancy for each site (due to excessive disorder, or part of the site overlapping with squeezed areas) and no attempts were made to match occupancies. Uij components of ADPs for disordered atoms closer to each other than 2.0 Angstrom were restrained to be similar. Water H atom positions were refined and O-H and H···H distances were restrained to 0.84 (2) and 1.36 (2) Angstrom, respectively, while a damping factor was applied. Some water H atom positions were further restrained based on hydrogen bonding considerations. In the final refinement cycles H atoms with low occupancies were constrained to ride on their carrier atoms and the damping factor was removed. Subject to these conditions the occupancy rates refined to the values given in the tables of the cif file.

### Fractional atomic coordinates and isotropic or equivalent isotropic displacement parameters (Å^2^)

**Table:**

	*x*	*y*	*z*	*U*_iso_*/*U*_eq_	Occ. (<1)
In1	0.42730 (2)	0.51469 (2)	0.18905 (2)	0.03389 (5)	
In2	0.56369 (2)	0.53389 (2)	0.37738 (2)	0.03459 (5)	
O1	0.34520 (17)	0.49088 (14)	0.08522 (13)	0.0415 (4)	
O2	0.26988 (17)	0.63287 (13)	0.20040 (13)	0.0397 (4)	
O3	0.0717 (2)	0.64950 (16)	0.01075 (15)	0.0497 (5)	
H3O	0.020516	0.657664	−0.019345	0.075*	
O4	0.57140 (17)	0.45100 (14)	0.25976 (13)	0.0455 (5)	
O5	0.52816 (17)	0.37557 (13)	0.12922 (13)	0.0411 (4)	
O6	0.8253 (2)	0.21109 (16)	0.20886 (15)	0.0537 (6)	
H6O	0.896623	0.181703	0.205605	0.081*	
O7	0.59121 (15)	0.52804 (14)	0.52133 (12)	0.0364 (4)	
O8	0.71809 (16)	0.41493 (14)	0.38087 (12)	0.0388 (4)	
O9	0.90123 (17)	0.43199 (16)	0.57286 (15)	0.0472 (5)	
H9O	0.950869	0.432237	0.602515	0.071*	
O10	0.44803 (17)	0.60840 (13)	0.28938 (13)	0.0399 (4)	
O11	0.46380 (17)	0.67505 (13)	0.43780 (14)	0.0414 (4)	
O12	0.2284 (2)	0.86105 (14)	0.30704 (15)	0.0524 (6)	
H12O	0.202009	0.912161	0.277748	0.079*	
O13	0.5494 (2)	0.59144 (16)	0.07719 (14)	0.0476 (5)	
H13B	0.604 (3)	0.609 (3)	0.088 (3)	0.071*	
H13A	0.576 (3)	0.568 (3)	0.0229 (18)	0.071*	
O14	0.30830 (18)	0.43647 (15)	0.30866 (14)	0.0427 (4)	
H14A	0.336 (3)	0.3795 (17)	0.330 (3)	0.064*	
H14B	0.237 (2)	0.435 (3)	0.309 (3)	0.064*	
O15	0.7033 (2)	0.6145 (2)	0.2944 (3)	0.0810 (10)	
H15A	0.680 (4)	0.641 (4)	0.247 (3)	0.122*	
H15B	0.7781 (19)	0.593 (4)	0.276 (4)	0.122*	
N5	0.1343 (10)	1.0732 (13)	0.1845 (15)	0.050 (3)	0.443 (7)
O16	0.2485 (8)	1.0514 (7)	0.1561 (7)	0.087 (3)	0.443 (7)
O17	0.0762 (6)	1.0116 (4)	0.2364 (4)	0.0621 (18)	0.443 (7)
O18	0.0769 (9)	1.1489 (8)	0.1526 (7)	0.062 (2)	0.443 (7)
N5B	0.1096 (8)	1.0702 (11)	0.1851 (13)	0.051 (2)	0.557 (7)
O16B	0.2066 (5)	1.0146 (4)	0.1890 (4)	0.0578 (14)	0.557 (7)
O17B	0.0105 (4)	1.0635 (3)	0.2483 (3)	0.0569 (13)	0.557 (7)
O18B	0.1139 (9)	1.1390 (6)	0.1223 (5)	0.0686 (19)	0.557 (7)
N6	0.9957 (2)	0.50130 (19)	0.2633 (2)	0.0473 (5)	
O19	1.10061 (19)	0.45204 (17)	0.25405 (17)	0.0524 (5)	
O20	0.9447 (2)	0.55804 (16)	0.32832 (18)	0.0528 (5)	
O21	0.9408 (2)	0.4925 (2)	0.2063 (2)	0.0668 (7)	
O23	0.3259 (5)	0.2728 (4)	0.1487 (5)	0.129 (3)	0.792 (11)
H23A	0.317472	0.252966	0.095333	0.194*	0.792 (11)
H23B	0.396276	0.292362	0.131928	0.194*	0.792 (11)
O22	0.9121 (4)	0.6555 (3)	−0.0832 (3)	0.0795 (12)	0.886 (7)
H22A	0.901 (6)	0.706 (2)	−0.117 (4)	0.119*	0.886 (7)
H22B	0.942 (6)	0.608 (3)	−0.115 (4)	0.119*	0.886 (7)
O24	0.7903 (5)	0.5624 (5)	0.0717 (4)	0.0820 (19)	0.590 (7)
H24A	0.754 (7)	0.611 (5)	0.107 (6)	0.123*	0.590 (7)
H24B	0.848 (6)	0.533 (5)	0.094 (6)	0.123*	0.590 (7)
O25	0.616 (2)	0.7286 (18)	0.1395 (18)	0.084 (6)	0.152 (9)
H25A	0.630387	0.766202	0.171871	0.127*	0.152 (9)
H25B	0.591205	0.762305	0.095684	0.127*	0.152 (9)
O22B	0.9010 (14)	0.5910 (19)	−0.0361 (17)	0.077 (5)	0.114 (7)
H22C	0.938459	0.577782	−0.093868	0.115*	0.114 (7)
H22D	0.878384	0.540475	−0.005618	0.115*	0.114 (7)
O24B	0.6901 (10)	0.6830 (7)	0.1027 (6)	0.080 (3)	0.410 (7)
H24C	0.764 (4)	0.663 (4)	0.069 (3)	0.121*	0.410 (7)
H24D	0.684 (6)	0.737 (5)	0.123 (10)	0.121*	0.410 (7)
O26	0.4029 (5)	0.2921 (3)	0.4115 (5)	0.0826 (13)	0.652 (5)
H26A	0.426 (8)	0.237 (3)	0.392 (7)	0.124*	0.652 (5)
H26B	0.446 (7)	0.303 (6)	0.443 (5)	0.124*	0.652 (5)
O27	0.4387 (17)	0.1825 (12)	0.3027 (13)	0.0840 (15)	0.161 (6)
H27A	0.512426	0.171499	0.267040	0.126*	0.161 (6)
H27B	0.398262	0.213590	0.264719	0.126*	0.161 (6)
O26B	0.4126 (9)	0.2528 (6)	0.3243 (8)	0.0840 (13)	0.348 (5)
H26C	0.420769	0.212829	0.370989	0.126*	0.348 (5)
H26D	0.368061	0.234118	0.298873	0.126*	0.348 (5)
O27B	0.3779 (17)	0.2429 (11)	0.4890 (15)	0.0829 (14)	0.179 (7)
H27C	0.315208	0.282720	0.482494	0.124*	0.179 (7)
H27D	0.421500	0.276216	0.501045	0.124*	0.179 (7)
N1	0.2471 (2)	0.56338 (15)	0.08175 (14)	0.0372 (5)	
H1N	0.209366	0.564206	0.039015	0.045*	
N2	0.6447 (2)	0.36642 (16)	0.22369 (14)	0.0398 (5)	
H2N	0.706562	0.337010	0.244564	0.048*	
N3	0.70532 (17)	0.47275 (16)	0.51926 (14)	0.0365 (4)	
H3N	0.736818	0.474859	0.565283	0.044*	
N4	0.37305 (19)	0.69352 (14)	0.32306 (15)	0.0360 (4)	
H4N	0.318985	0.725950	0.295052	0.043*	
C1	0.2100 (2)	0.63161 (17)	0.14285 (16)	0.0336 (5)	
C2	0.0970 (2)	0.70401 (17)	0.14686 (17)	0.0334 (5)	
C3	0.0299 (2)	0.71124 (19)	0.08285 (19)	0.0377 (5)	
C4	−0.0770 (3)	0.7813 (2)	0.0930 (2)	0.0451 (6)	
H4	−0.121786	0.786790	0.048854	0.054*	
C5	−0.1177 (3)	0.8425 (2)	0.1671 (2)	0.0491 (7)	
H5	−0.191096	0.889457	0.174141	0.059*	
C6	−0.0528 (3)	0.8362 (2)	0.2313 (2)	0.0506 (7)	
H6	−0.081218	0.878680	0.282113	0.061*	
C7	0.0541 (2)	0.7674 (2)	0.2208 (2)	0.0421 (6)	
H7	0.098971	0.763232	0.264669	0.051*	
C8	0.6192 (2)	0.33083 (17)	0.15665 (16)	0.0344 (5)	
C9	0.6919 (2)	0.23985 (17)	0.11285 (16)	0.0342 (5)	
C10	0.7905 (2)	0.18173 (18)	0.13967 (18)	0.0390 (5)	
C11	0.8506 (3)	0.09571 (19)	0.0951 (2)	0.0462 (6)	
H11	0.916324	0.055457	0.114215	0.055*	
C12	0.8157 (3)	0.0686 (2)	0.0237 (2)	0.0495 (7)	
H12	0.857088	0.009470	−0.005507	0.059*	
C13	0.7206 (3)	0.1266 (2)	−0.0061 (2)	0.0479 (7)	
H13	0.698139	0.108430	−0.056489	0.058*	
C14	0.6591 (3)	0.21151 (19)	0.03901 (19)	0.0402 (5)	
H14	0.593415	0.251208	0.019449	0.048*	
C15	0.7656 (2)	0.41697 (18)	0.44685 (16)	0.0335 (5)	
C16	0.8865 (2)	0.35688 (17)	0.44379 (16)	0.0324 (4)	
C17	0.9511 (2)	0.36547 (18)	0.50577 (18)	0.0350 (5)	
C18	1.0662 (2)	0.3068 (2)	0.4972 (2)	0.0418 (6)	
H18	1.109663	0.312668	0.539478	0.050*	
C19	1.1165 (3)	0.2406 (2)	0.4275 (2)	0.0471 (7)	
H19	1.194922	0.201193	0.421513	0.057*	
C20	1.0535 (3)	0.2312 (2)	0.3664 (2)	0.0489 (7)	
H20	1.088423	0.184911	0.318902	0.059*	
C21	0.9390 (2)	0.28914 (19)	0.3739 (2)	0.0401 (5)	
H21	0.896402	0.282595	0.331230	0.048*	
C22	0.3849 (2)	0.72507 (17)	0.39896 (18)	0.0333 (5)	
C23	0.3071 (2)	0.81570 (16)	0.43989 (17)	0.0323 (4)	
C24	0.2326 (2)	0.88216 (18)	0.39293 (18)	0.0376 (5)	
C25	0.1637 (3)	0.96712 (19)	0.4359 (2)	0.0463 (6)	
H25	0.113587	1.012406	0.404322	0.056*	
C26	0.1680 (3)	0.9858 (2)	0.5240 (2)	0.0483 (7)	
H26	0.121024	1.044132	0.552495	0.058*	
C27	0.2395 (3)	0.9209 (2)	0.5714 (2)	0.0492 (7)	
H27	0.241017	0.933940	0.632569	0.059*	
C28	0.3092 (3)	0.83638 (19)	0.5292 (2)	0.0422 (6)	
H28	0.359128	0.791915	0.561565	0.051*	

### Atomic displacement parameters (Å^2^)

**Table:**

	*U*^11^	*U*^22^	*U*^33^	*U*^12^	*U*^13^	*U*^23^
In1	0.03514 (8)	0.03784 (9)	0.02316 (7)	0.01602 (6)	−0.01382 (6)	−0.01154 (6)
In2	0.03067 (8)	0.04148 (9)	0.02773 (8)	0.01479 (6)	−0.01486 (6)	−0.01243 (6)
O1	0.0444 (9)	0.0434 (9)	0.0328 (8)	0.0214 (8)	−0.0218 (7)	−0.0165 (7)
O2	0.0400 (9)	0.0417 (9)	0.0350 (8)	0.0175 (7)	−0.0206 (7)	−0.0160 (7)
O3	0.0522 (11)	0.0526 (11)	0.0476 (11)	0.0146 (9)	−0.0317 (9)	−0.0163 (9)
O4	0.0448 (10)	0.0502 (10)	0.0360 (9)	0.0284 (8)	−0.0237 (8)	−0.0214 (8)
O5	0.0433 (9)	0.0399 (9)	0.0370 (9)	0.0193 (7)	−0.0213 (7)	−0.0170 (7)
O6	0.0568 (12)	0.0521 (11)	0.0475 (11)	0.0304 (9)	−0.0305 (10)	−0.0225 (9)
O7	0.0269 (7)	0.0489 (10)	0.0306 (8)	0.0118 (7)	−0.0140 (6)	−0.0141 (7)
O8	0.0355 (8)	0.0481 (10)	0.0282 (8)	0.0148 (7)	−0.0158 (7)	−0.0117 (7)
O9	0.0339 (9)	0.0625 (12)	0.0457 (10)	0.0136 (8)	−0.0212 (8)	−0.0225 (9)
O10	0.0412 (9)	0.0390 (9)	0.0359 (8)	0.0202 (7)	−0.0204 (7)	−0.0172 (7)
O11	0.0380 (9)	0.0399 (9)	0.0504 (10)	0.0125 (7)	−0.0267 (8)	−0.0182 (8)
O12	0.0741 (14)	0.0380 (10)	0.0460 (11)	0.0218 (9)	−0.0365 (10)	−0.0166 (8)
O13	0.0570 (12)	0.0515 (11)	0.0289 (8)	0.0010 (9)	−0.0093 (8)	−0.0154 (8)
O14	0.0397 (9)	0.0436 (10)	0.0396 (9)	0.0078 (8)	−0.0152 (8)	−0.0061 (8)
O15	0.0360 (11)	0.0768 (18)	0.109 (2)	−0.0005 (12)	−0.0161 (13)	0.0296 (17)
N5	0.068 (4)	0.042 (4)	0.041 (3)	0.019 (4)	−0.033 (4)	−0.021 (3)
O16	0.074 (5)	0.080 (5)	0.092 (5)	0.030 (4)	−0.035 (4)	−0.012 (4)
O17	0.078 (4)	0.046 (3)	0.061 (3)	0.018 (3)	−0.039 (3)	−0.004 (2)
O18	0.066 (5)	0.057 (4)	0.056 (4)	0.023 (3)	−0.031 (3)	−0.009 (3)
N5B	0.071 (4)	0.041 (3)	0.047 (3)	0.013 (3)	−0.038 (3)	−0.017 (2)
O16B	0.062 (3)	0.050 (3)	0.048 (3)	0.024 (2)	−0.021 (2)	−0.0088 (19)
O17B	0.062 (3)	0.057 (3)	0.047 (2)	0.016 (2)	−0.0244 (19)	−0.0151 (18)
O18B	0.099 (6)	0.044 (3)	0.060 (4)	0.008 (3)	−0.040 (3)	0.006 (3)
N6	0.0432 (12)	0.0472 (13)	0.0572 (14)	−0.0131 (10)	−0.0234 (11)	0.0038 (11)
O19	0.0382 (10)	0.0595 (13)	0.0620 (13)	−0.0069 (9)	−0.0186 (9)	−0.0093 (10)
O20	0.0468 (11)	0.0440 (11)	0.0691 (14)	−0.0042 (9)	−0.0204 (10)	−0.0104 (10)
O21	0.0627 (15)	0.0757 (17)	0.0813 (18)	−0.0166 (13)	−0.0461 (14)	−0.0051 (14)
O23	0.115 (5)	0.108 (4)	0.145 (5)	−0.039 (3)	0.003 (4)	−0.023 (4)
O22	0.116 (3)	0.067 (2)	0.080 (2)	−0.005 (2)	−0.070 (2)	−0.0127 (17)
O24	0.064 (3)	0.103 (4)	0.090 (4)	−0.007 (3)	−0.043 (3)	−0.012 (3)
O25	0.084 (10)	0.092 (10)	0.071 (9)	−0.029 (8)	−0.012 (8)	0.002 (8)
O22B	0.116 (9)	0.055 (8)	0.086 (9)	−0.007 (8)	−0.067 (8)	−0.025 (7)
O24B	0.099 (6)	0.090 (6)	0.068 (5)	−0.055 (5)	−0.029 (4)	0.008 (4)
O26	0.086 (3)	0.053 (2)	0.120 (4)	−0.0028 (19)	−0.060 (3)	0.010 (2)
O27	0.087 (3)	0.052 (2)	0.121 (4)	−0.004 (2)	−0.056 (3)	0.008 (2)
O26B	0.087 (3)	0.053 (2)	0.121 (4)	−0.003 (2)	−0.057 (3)	0.009 (2)
O27B	0.086 (3)	0.053 (2)	0.120 (4)	−0.004 (2)	−0.059 (3)	0.011 (2)
N1	0.0410 (10)	0.0378 (10)	0.0308 (9)	0.0148 (8)	−0.0201 (8)	−0.0117 (8)
N2	0.0401 (10)	0.0412 (11)	0.0310 (9)	0.0238 (9)	−0.0176 (8)	−0.0174 (8)
N3	0.0269 (8)	0.0497 (12)	0.0298 (9)	0.0117 (8)	−0.0148 (7)	−0.0108 (8)
N4	0.0386 (10)	0.0319 (9)	0.0348 (9)	0.0152 (8)	−0.0182 (8)	−0.0137 (7)
C1	0.0346 (11)	0.0348 (11)	0.0279 (10)	0.0091 (9)	−0.0142 (8)	−0.0064 (8)
C2	0.0311 (10)	0.0307 (10)	0.0344 (11)	0.0068 (8)	−0.0133 (9)	−0.0035 (8)
C3	0.0365 (12)	0.0359 (12)	0.0383 (12)	0.0048 (9)	−0.0168 (10)	−0.0022 (9)
C4	0.0380 (13)	0.0443 (14)	0.0523 (15)	0.0070 (11)	−0.0236 (12)	−0.0028 (12)
C5	0.0379 (13)	0.0392 (13)	0.0653 (18)	0.0128 (11)	−0.0217 (13)	−0.0082 (12)
C6	0.0438 (14)	0.0413 (14)	0.0607 (18)	0.0141 (11)	−0.0172 (13)	−0.0196 (13)
C7	0.0375 (12)	0.0405 (13)	0.0454 (14)	0.0117 (10)	−0.0176 (11)	−0.0142 (11)
C8	0.0367 (11)	0.0350 (11)	0.0243 (9)	0.0138 (9)	−0.0114 (8)	−0.0096 (8)
C9	0.0362 (11)	0.0305 (10)	0.0279 (10)	0.0116 (8)	−0.0093 (8)	−0.0089 (8)
C10	0.0407 (12)	0.0351 (11)	0.0326 (11)	0.0146 (9)	−0.0119 (9)	−0.0100 (9)
C11	0.0463 (14)	0.0335 (12)	0.0506 (15)	0.0165 (10)	−0.0166 (12)	−0.0139 (11)
C12	0.0520 (16)	0.0344 (12)	0.0542 (16)	0.0121 (11)	−0.0127 (13)	−0.0206 (11)
C13	0.0516 (15)	0.0416 (14)	0.0481 (15)	0.0087 (12)	−0.0170 (12)	−0.0211 (12)
C14	0.0435 (13)	0.0363 (12)	0.0370 (12)	0.0088 (10)	−0.0145 (10)	−0.0135 (9)
C15	0.0279 (10)	0.0408 (12)	0.0268 (9)	0.0070 (8)	−0.0107 (8)	−0.0043 (8)
C16	0.0264 (9)	0.0357 (11)	0.0292 (10)	0.0064 (8)	−0.0099 (8)	−0.0007 (8)
C17	0.0277 (10)	0.0381 (12)	0.0354 (11)	0.0037 (8)	−0.0119 (9)	−0.0019 (9)
C18	0.0328 (11)	0.0427 (13)	0.0508 (15)	0.0060 (10)	−0.0215 (11)	−0.0078 (11)
C19	0.0362 (12)	0.0377 (13)	0.0666 (18)	0.0110 (10)	−0.0236 (12)	−0.0127 (12)
C20	0.0397 (13)	0.0424 (14)	0.0622 (18)	0.0161 (11)	−0.0220 (13)	−0.0212 (13)
C21	0.0351 (11)	0.0402 (13)	0.0432 (13)	0.0086 (10)	−0.0176 (10)	−0.0109 (10)
C22	0.0323 (10)	0.0311 (10)	0.0375 (11)	0.0046 (8)	−0.0165 (9)	−0.0094 (9)
C23	0.0315 (10)	0.0282 (10)	0.0367 (11)	0.0036 (8)	−0.0128 (9)	−0.0112 (8)
C24	0.0429 (13)	0.0304 (11)	0.0373 (12)	0.0075 (9)	−0.0165 (10)	−0.0096 (9)
C25	0.0543 (16)	0.0322 (12)	0.0486 (15)	0.0128 (11)	−0.0211 (12)	−0.0134 (10)
C26	0.0580 (17)	0.0335 (12)	0.0475 (15)	0.0093 (11)	−0.0149 (13)	−0.0179 (11)
C27	0.0656 (18)	0.0388 (13)	0.0438 (14)	0.0059 (12)	−0.0231 (13)	−0.0174 (11)
C28	0.0513 (15)	0.0362 (12)	0.0427 (13)	0.0037 (11)	−0.0239 (12)	−0.0121 (10)

### Geometric parameters (Å, º)

**Table:**

In1—O13	2.156 (2)	O27—H27A	0.8477
In1—O1	2.1594 (17)	O27—H27B	0.8627
In1—O2	2.1774 (16)	O26B—H26C	0.8430
In1—O5	2.1930 (16)	O26B—H26D	0.8446
In1—O14	2.205 (2)	O27B—H27C	0.8468
In1—O10	2.2254 (18)	O27B—H27D	0.8479
In1—O4	2.2348 (17)	N1—C1	1.314 (3)
In2—O15	2.167 (3)	N1—H1N	0.8800
In2—O10	2.1851 (16)	N2—C8	1.314 (3)
In2—O8	2.1915 (16)	N2—H2N	0.8800
In2—O4	2.1916 (18)	N3—C15	1.320 (3)
In2—O11	2.2179 (17)	N3—H3N	0.8800
In2—O7^i^	2.2228 (19)	N4—C22	1.318 (3)
In2—O7	2.2312 (16)	N4—H4N	0.8800
O1—N1	1.372 (2)	C1—C2	1.476 (3)
O2—C1	1.274 (3)	C2—C7	1.395 (3)
O3—C3	1.361 (3)	C2—C3	1.397 (3)
O3—H3O	0.8400	C3—C4	1.395 (3)
O4—N2	1.368 (2)	C4—C5	1.378 (4)
O5—C8	1.274 (3)	C4—H4	0.9500
O6—C10	1.361 (3)	C5—C6	1.381 (4)
O6—H6O	0.8400	C5—H5	0.9500
O7—N3	1.389 (2)	C6—C7	1.386 (3)
O8—C15	1.278 (3)	C6—H6	0.9500
O9—C17	1.354 (3)	C7—H7	0.9500
O9—H9O	0.8400	C8—C9	1.476 (3)
O10—N4	1.370 (2)	C9—C10	1.401 (3)
O11—C22	1.277 (3)	C9—C14	1.402 (4)
O12—C24	1.356 (3)	C10—C11	1.394 (3)
O12—H12O	0.8400	C11—C12	1.376 (4)
O13—H13B	0.817 (18)	C11—H11	0.9500
O13—H13A	0.842 (18)	C12—C13	1.389 (4)
O14—H14A	0.857 (17)	C12—H12	0.9500
O14—H14B	0.843 (17)	C13—C14	1.387 (3)
O15—H15A	0.835 (19)	C13—H13	0.9500
O15—H15B	0.829 (19)	C14—H14	0.9500
N5—O17	1.251 (12)	C15—C16	1.477 (3)
N5—O18	1.252 (11)	C16—C21	1.396 (3)
N5—O16	1.253 (11)	C16—C17	1.401 (3)
N5B—O18B	1.245 (9)	C17—C18	1.398 (3)
N5B—O16B	1.247 (9)	C18—C19	1.378 (4)
N5B—O17B	1.248 (10)	C18—H18	0.9500
N6—O20	1.240 (4)	C19—C20	1.380 (4)
N6—O19	1.250 (3)	C19—H19	0.9500
N6—O21	1.252 (3)	C20—C21	1.391 (3)
O23—H23A	0.9121	C20—H20	0.9500
O23—H23B	0.8870	C21—H21	0.9500
O22—H22A	0.824 (19)	C22—C23	1.473 (3)
O22—H22B	0.821 (19)	C23—C28	1.393 (3)
O24—H24A	0.86 (2)	C23—C24	1.406 (3)
O24—H24B	0.85 (2)	C24—C25	1.393 (3)
O25—H25A	0.8399	C25—C26	1.376 (4)
O25—H25B	0.8397	C25—H25	0.9500
O22B—H22C	0.8432	C26—C27	1.377 (4)
O22B—H22D	0.8417	C26—H26	0.9500
O24B—H24C	0.85 (2)	C27—C28	1.385 (4)
O24B—H24D	0.84 (2)	C27—H27	0.9500
O26—H26A	0.83 (2)	C28—H28	0.9500
O26—H26B	0.843 (16)		
			
O13—In1—O1	89.83 (8)	C8—N2—H2N	121.3
O13—In1—O2	93.89 (8)	O4—N2—H2N	121.3
O1—In1—O2	74.33 (6)	C15—N3—O7	118.69 (18)
O13—In1—O5	91.12 (8)	C15—N3—H3N	120.7
O1—In1—O5	73.42 (6)	O7—N3—H3N	120.7
O2—In1—O5	147.32 (7)	C22—N4—O10	117.54 (18)
O13—In1—O14	176.88 (8)	C22—N4—H4N	121.2
O1—In1—O14	93.25 (8)	O10—N4—H4N	121.2
O2—In1—O14	87.42 (7)	O2—C1—N1	119.66 (19)
O5—In1—O14	89.29 (8)	O2—C1—C2	120.4 (2)
O13—In1—O10	85.39 (8)	N1—C1—C2	119.9 (2)
O1—In1—O10	151.22 (6)	C7—C2—C3	118.6 (2)
O2—In1—O10	77.70 (6)	C7—C2—C1	117.3 (2)
O5—In1—O10	134.94 (6)	C3—C2—C1	124.1 (2)
O14—In1—O10	92.13 (8)	O3—C3—C4	121.3 (2)
O13—In1—O4	91.89 (9)	O3—C3—C2	118.7 (2)
O1—In1—O4	144.16 (6)	C4—C3—C2	120.0 (2)
O2—In1—O4	141.12 (6)	C5—C4—C3	120.1 (3)
O5—In1—O4	70.76 (6)	C5—C4—H4	120.0
O14—In1—O4	85.32 (8)	C3—C4—H4	120.0
O10—In1—O4	64.50 (6)	C4—C5—C6	120.7 (2)
O15—In2—O10	89.73 (10)	C4—C5—H5	119.7
O15—In2—O8	84.54 (9)	C6—C5—H5	119.7
O10—In2—O8	143.67 (6)	C5—C6—C7	119.4 (3)
O15—In2—O4	96.99 (13)	C5—C6—H6	120.3
O10—In2—O4	65.88 (6)	C7—C6—H6	120.3
O8—In2—O4	79.23 (6)	C6—C7—C2	121.2 (3)
O15—In2—O11	84.31 (11)	C6—C7—H7	119.4
O10—In2—O11	71.51 (6)	C2—C7—H7	119.4
O8—In2—O11	142.83 (6)	O5—C8—N2	119.10 (19)
O4—In2—O11	137.36 (6)	O5—C8—C9	118.9 (2)
O15—In2—O7^i^	170.29 (11)	N2—C8—C9	122.0 (2)
O10—In2—O7^i^	90.28 (7)	C10—C9—C14	118.8 (2)
O8—In2—O7^i^	101.08 (7)	C10—C9—C8	124.5 (2)
O4—In2—O7^i^	91.88 (8)	C14—C9—C8	116.7 (2)
O11—In2—O7^i^	86.49 (7)	O6—C10—C11	121.6 (2)
O15—In2—O7	98.35 (11)	O6—C10—C9	118.8 (2)
O10—In2—O7	143.60 (6)	C11—C10—C9	119.5 (2)
O8—In2—O7	72.67 (6)	C12—C11—C10	120.6 (2)
O4—In2—O7	146.36 (7)	C12—C11—H11	119.7
O11—In2—O7	74.06 (6)	C10—C11—H11	119.7
O7^i^—In2—O7	76.08 (7)	C11—C12—C13	120.8 (2)
N1—O1—In1	111.75 (13)	C11—C12—H12	119.6
C1—O2—In1	114.73 (14)	C13—C12—H12	119.6
C3—O3—H3O	109.5	C14—C13—C12	118.9 (3)
N2—O4—In2	132.33 (13)	C14—C13—H13	120.5
N2—O4—In1	114.14 (13)	C12—C13—H13	120.5
In2—O4—In1	113.49 (7)	C13—C14—C9	121.3 (2)
C8—O5—In1	118.44 (15)	C13—C14—H14	119.4
C10—O6—H6O	109.5	C9—C14—H14	119.4
N3—O7—In2^i^	113.96 (15)	O8—C15—N3	119.61 (19)
N3—O7—In2	110.00 (12)	O8—C15—C16	120.8 (2)
In2^i^—O7—In2	103.92 (7)	N3—C15—C16	119.5 (2)
C15—O8—In2	114.99 (14)	C21—C16—C17	118.9 (2)
C17—O9—H9O	109.5	C21—C16—C15	117.4 (2)
N4—O10—In2	114.93 (13)	C17—C16—C15	123.7 (2)
N4—O10—In1	127.85 (14)	O9—C17—C18	120.7 (2)
In2—O10—In1	114.12 (7)	O9—C17—C16	119.1 (2)
C22—O11—In2	116.63 (15)	C18—C17—C16	120.2 (2)
C24—O12—H12O	109.5	C19—C18—C17	120.0 (2)
In1—O13—H13B	120 (3)	C19—C18—H18	120.0
In1—O13—H13A	115 (3)	C17—C18—H18	120.0
H13B—O13—H13A	110 (3)	C18—C19—C20	120.4 (2)
In1—O14—H14A	120 (2)	C18—C19—H19	119.8
In1—O14—H14B	120 (3)	C20—C19—H19	119.8
H14A—O14—H14B	105 (2)	C19—C20—C21	120.3 (3)
In2—O15—H15A	105 (4)	C19—C20—H20	119.8
In2—O15—H15B	127 (4)	C21—C20—H20	119.8
H15A—O15—H15B	110 (3)	C20—C21—C16	120.3 (2)
O17—N5—O18	118.9 (11)	C20—C21—H21	119.9
O17—N5—O16	118.6 (11)	C16—C21—H21	119.9
O18—N5—O16	121.8 (11)	O11—C22—N4	119.0 (2)
O18B—N5B—O16B	119.0 (9)	O11—C22—C23	120.2 (2)
O18B—N5B—O17B	119.5 (9)	N4—C22—C23	120.8 (2)
O16B—N5B—O17B	121.1 (9)	C28—C23—C24	118.8 (2)
O20—N6—O19	121.3 (3)	C28—C23—C22	117.8 (2)
O20—N6—O21	119.4 (3)	C24—C23—C22	123.3 (2)
O19—N6—O21	119.3 (3)	O12—C24—C25	121.6 (2)
H23A—O23—H23B	108.5	O12—C24—C23	118.9 (2)
H22A—O22—H22B	112 (3)	C25—C24—C23	119.5 (2)
H24A—O24—H24B	105 (3)	C26—C25—C24	120.3 (2)
H25A—O25—H25B	107.9	C26—C25—H25	119.8
H22C—O22B—H22D	107.4	C24—C25—H25	119.8
H24C—O24B—H24D	109 (3)	C25—C26—C27	120.8 (2)
H26A—O26—H26B	112 (3)	C25—C26—H26	119.6
H27A—O27—H27B	104.7	C27—C26—H26	119.6
H26C—O26B—H26D	107.9	C26—C27—C28	119.5 (3)
H27C—O27B—H27D	106.0	C26—C27—H27	120.2
C1—N1—O1	119.36 (19)	C28—C27—H27	120.2
C1—N1—H1N	120.3	C27—C28—C23	120.9 (2)
O1—N1—H1N	120.3	C27—C28—H28	119.5
C8—N2—O4	117.36 (18)	C23—C28—H28	119.5
			
In1—O1—N1—C1	−4.7 (3)	C12—C13—C14—C9	0.6 (5)
In2—O4—N2—C8	174.4 (2)	C10—C9—C14—C13	1.3 (4)
In1—O4—N2—C8	−3.1 (3)	C8—C9—C14—C13	−179.2 (3)
In2^i^—O7—N3—C15	101.5 (2)	In2—O8—C15—N3	15.6 (3)
In2—O7—N3—C15	−14.7 (3)	In2—O8—C15—C16	−165.05 (18)
In2—O10—N4—C22	5.2 (3)	O7—N3—C15—O8	−0.1 (4)
In1—O10—N4—C22	163.91 (19)	O7—N3—C15—C16	−179.4 (2)
In1—O2—C1—N1	−1.4 (3)	O8—C15—C16—C21	−8.2 (4)
In1—O2—C1—C2	176.31 (18)	N3—C15—C16—C21	171.1 (3)
O1—N1—C1—O2	4.2 (4)	O8—C15—C16—C17	170.8 (2)
O1—N1—C1—C2	−173.5 (2)	N3—C15—C16—C17	−9.9 (4)
O2—C1—C2—C7	−5.3 (4)	C21—C16—C17—O9	179.1 (3)
N1—C1—C2—C7	172.4 (3)	C15—C16—C17—O9	0.2 (4)
O2—C1—C2—C3	176.1 (3)	C21—C16—C17—C18	0.0 (4)
N1—C1—C2—C3	−6.2 (4)	C15—C16—C17—C18	−179.0 (3)
C7—C2—C3—O3	180.0 (3)	O9—C17—C18—C19	−178.9 (3)
C1—C2—C3—O3	−1.4 (4)	C16—C17—C18—C19	0.2 (4)
C7—C2—C3—C4	0.5 (4)	C17—C18—C19—C20	−0.5 (5)
C1—C2—C3—C4	179.1 (3)	C18—C19—C20—C21	0.6 (5)
O3—C3—C4—C5	179.6 (3)	C19—C20—C21—C16	−0.4 (5)
C2—C3—C4—C5	−1.0 (5)	C17—C16—C21—C20	0.1 (4)
C3—C4—C5—C6	0.8 (5)	C15—C16—C21—C20	179.2 (3)
C4—C5—C6—C7	−0.1 (5)	In2—O11—C22—N4	−4.1 (3)
C5—C6—C7—C2	−0.3 (5)	In2—O11—C22—C23	174.78 (18)
C3—C2—C7—C6	0.1 (4)	O10—N4—C22—O11	−0.7 (4)
C1—C2—C7—C6	−178.5 (3)	O10—N4—C22—C23	−179.6 (2)
In1—O5—C8—N2	4.1 (3)	O11—C22—C23—C28	−9.8 (4)
In1—O5—C8—C9	−176.65 (18)	N4—C22—C23—C28	169.1 (3)
O4—N2—C8—O5	−0.5 (4)	O11—C22—C23—C24	169.5 (3)
O4—N2—C8—C9	−179.8 (2)	N4—C22—C23—C24	−11.6 (4)
O5—C8—C9—C10	−177.1 (3)	C28—C23—C24—O12	−178.5 (3)
N2—C8—C9—C10	2.2 (4)	C22—C23—C24—O12	2.2 (4)
O5—C8—C9—C14	3.5 (4)	C28—C23—C24—C25	0.6 (4)
N2—C8—C9—C14	−177.2 (3)	C22—C23—C24—C25	−178.7 (3)
C14—C9—C10—O6	177.6 (3)	O12—C24—C25—C26	178.7 (3)
C8—C9—C10—O6	−1.9 (4)	C23—C24—C25—C26	−0.3 (5)
C14—C9—C10—C11	−2.3 (4)	C24—C25—C26—C27	−0.4 (5)
C8—C9—C10—C11	178.2 (3)	C25—C26—C27—C28	0.9 (5)
O6—C10—C11—C12	−178.5 (3)	C26—C27—C28—C23	−0.6 (5)
C9—C10—C11—C12	1.4 (5)	C24—C23—C28—C27	−0.1 (4)
C10—C11—C12—C13	0.6 (5)	C22—C23—C28—C27	179.2 (3)
C11—C12—C13—C14	−1.6 (5)		

Symmetry code: (i) −*x*+1, −*y*+1, −*z*+1.

### Hydrogen-bond geometry (Å, º)

**Table:**

*D*—H···*A*	*D*—H	H···*A*	*D*···*A*	*D*—H···*A*
O3—H3*O*···O22^ii^	0.84	1.83	2.664 (4)	171
O3—H3*O*···O22*B*^ii^	0.84	1.97	2.668 (17)	140
O6—H6*O*···O18^iii^	0.84	1.98	2.775 (10)	157
O6—H6*O*···O17*B*^iii^	0.84	2.06	2.810 (5)	149
O9—H9*O*···O20^iv^	0.84	1.88	2.717 (3)	176
O12—H12*O*···O17	0.84	1.99	2.778 (5)	156
O12—H12*O*···O16*B*	0.84	1.81	2.609 (6)	158
O13—H13*B*···O24	0.82 (2)	2.09 (3)	2.761 (6)	140 (4)
O13—H13*B*···O25	0.82 (2)	2.01 (4)	2.64 (3)	134 (4)
O13—H13*B*···O24*B*	0.82 (2)	1.70 (2)	2.485 (7)	160 (5)
O13—H13*A*···O1^v^	0.84 (2)	1.77 (2)	2.601 (2)	170 (4)
O14—H14*A*···O26	0.86 (2)	1.83 (2)	2.631 (5)	154 (4)
O14—H14*A*···O26*B*	0.86 (2)	1.82 (2)	2.629 (8)	158 (4)
O14—H14*B*···O19^ii^	0.84 (2)	1.99 (2)	2.782 (3)	157 (4)
O15—H15*A*···O25	0.84 (2)	2.11 (3)	2.93 (3)	171 (6)
O15—H15*A*···O24*B*	0.84 (2)	2.10 (3)	2.891 (9)	158 (5)
O15—H15*B*···O20	0.83 (2)	2.27 (3)	2.970 (4)	142 (5)
O15—H15*B*···O21	0.83 (2)	2.16 (3)	2.924 (4)	154 (6)
O23—H23*B*···O5	0.89	2.16	3.003 (6)	160
O22—H22*A*···O18^vi^	0.82 (2)	2.11 (4)	2.842 (12)	148 (6)
O22—H22*B*···O21^vii^	0.82 (2)	2.03 (3)	2.832 (5)	164 (7)
O24—H24*A*···O25	0.86 (2)	2.03 (2)	2.80 (3)	149 (6)
O24—H24*B*···O21	0.85 (2)	2.21 (4)	3.003 (5)	155 (6)
O22*B*—H22*C*···O21^vii^	0.84	2.00	2.808 (16)	159
O24*B*—H24*C*···O22*B*	0.85 (2)	2.02 (2)	2.824 (19)	156 (7)
O26—H26*A*···O27	0.83 (2)	1.56 (6)	2.259 (16)	140 (9)
O27—H27*B*···O23	0.86	2.14	2.987 (19)	168
O26*B*—H26*C*···O27*B*	0.84	1.73	2.31 (2)	124
O26*B*—H26*D*···O23	0.84	2.38	3.033 (13)	134
O27*B*—H27*D*···O11^i^	0.85	2.13	2.955 (18)	163
N1—H1*N*···O3	0.88	1.92	2.605 (3)	134
N2—H2*N*···O6	0.88	2.02	2.669 (2)	130
N2—H2*N*···O8	0.88	2.48	2.935 (3)	113
N3—H3*N*···O9	0.88	1.94	2.605 (3)	132
N3—H3*N*···O14^i^	0.88	2.24	2.939 (3)	137
N4—H4*N*···O2	0.88	2.33	2.804 (3)	114
N4—H4*N*···O12	0.88	1.97	2.621 (3)	130

Symmetry codes: (i) −*x*+1, −*y*+1, −*z*+1; (ii) *x*−1, *y*, *z*; (iii) *x*+1, *y*−1, *z*; (iv) −*x*+2, −*y*+1, −*z*+1; (v) −*x*+1, −*y*+1, −*z*; (vi) −*x*+1, −*y*+2, −*z*; (vii) −*x*+2, −*y*+1, −*z*.
